# Knowledge, attitudes and practices on tuberculosis transmission and prevention among auxiliary healthcare professionals in three Brazilian high-burden cities: a cross-sectional survey

**DOI:** 10.1186/s12913-019-4231-x

**Published:** 2019-07-30

**Authors:** Anete Trajman, Maria F. Wakoff-Pereira, Jonas Ramos-Silva, Marcelo Cordeiro-Santos, Maria de Fátima Militão de Albuquerque, Philip C. Hill, Dick Menzies

**Affiliations:** 1 Programa de Pós-graduação em Clínica Médica, Universidade Federal do Rio de Janeiro, Universidade, Rio de Janeiro, Rua Macedo Sobrinho 74/203, Humaitá, Rio de Janeiro, 22271-080 Brazil; 2 Respiratory Epidemiology & Clinical Research Unit (RECRU), McGill University, Montreal, Canada; 3 Fundação de Medicina Tropical Dr. Heitor Dourado Vieira, Universidade Estadual do Amazonas, Manaus, AM Brazil; 4 Centro de Pesquisas Aggeu Magalhães, FioCruz, Recife, PE Brazil; 5 Centre for International Health, University of Otago, Dunedin, New Zealand

**Keywords:** Primary health care, Latent tuberculosis infection, Auxiliary healthcare worker, Health knowledge attitudes and practice survey

## Abstract

**Background:**

Tuberculosis elimination requires treatment of latently infected high-risk persons, such as contacts of index cases. Identification and referral of tuberculosis contacts for investigation are major barriers in the contact cascade-of-care. These tasks rely heavily on auxiliary primary healthcare workers in many low- and middle-income countries. We aimed to understand their knowledge, attitudes and practices (KAP) regarding contact investigation in Brazil.

**Methods:**

We conducted a cross-sectional KAP survey on tuberculosis transmission and prevention among 135 auxiliary healthcare workers in three tuberculosis high-burden Brazilian cities. Trained interviewers applied a translated version of a previously applied questionnaire. Open answers were classified in pre-defined objective categories and analysed quantitatively. Answers were further classified as satisfactory or not according to criteria set by a panel of three specialists.

**Results:**

Although 66% had received tuberculosis training in the past 10 years, only 19% were trained for tuberculosis prevention. 64% could not clearly distinguish latent tuberculosis infection (LTBI) from active tuberculosis; 63% did not know how to diagnose LTBI and 52% did not know how to prevent progression to active tuberculosis. Most believed that it is important to investigate adult (99%) and child (96%) contacts for LTBI. However, not all invite contacts - children (81%) or adults (71%) - to the clinic, despite only 24% perceiving difficulties for investigation.

**Conclusions:**

Gaps in KAP among auxiliary health workers have been reported in other areas, such as obstetrics and other infectious diseases. To the best of our knowledge, this is the first KAP survey on tuberculosis transmission and prevention among auxiliary health care workers, and relevant gaps were also found. Knowledge gaps were notably related to LTBI management, including how to recognize it and prevent progression to active tuberculosis through treatment, despite most recognizing the importance of investigating contacts. Auxiliary healthcare workers in three Brazilian high-burden cities have important knowledge gaps despite their perception of the importance of tuberculosis prevention among contacts. They need to incorporate contact referral as one of their tasks to enable progress toward the target of tuberculosis elimination.

**Electronic supplementary material:**

The online version of this article (10.1186/s12913-019-4231-x) contains supplementary material, which is available to authorized users.

## Background

Tuberculosis (TB) is one of the leading causes of death from infectious disease in the world, although effective prevention through treatment of latent TB infection (LTBI) has been available for more than half a century, which makes most of these deaths preventable [1]. One fourth of the world’s population has LTBI [2], which constitutes a reservoir for new TB cases. TB trend modelling projections have estimated that without tackling LTBI treatment, the End TB Strategy will not attain its goals [3]. Thus, TB index case contact tracing is an important task of primary health care (PHC) services. However, less than 10% of the people who need LTBI treatment will receive a prescription [4]. Barriers to healthcare access, patients’ and healthcare workers’ knowledge and beliefs are among possible explanations for losses in the contact cascade-of-care [4].

PHC is the cornerstone of universal health coverage [5], one of the Sustainable Development Goals [6], and is the usual setting for TB care. PHC actions in Brazil and other low and medium-income countries (LMIC) rely heavily on auxiliary health professionals [7, 8]. In Brazil, PHC is organized as the Family Health Strategy, which covers 2/3 of the Brazilian population [9], the largest public health system in the world. Coverage by the Family Health Strategy is significantly associated with successful treatment of active TB [10]. In this strategy, the so-called auxiliary “community health agents”, secondary level education workers who live in the community and are trained for health tasks [11], are responsible for home visits to detect those with respiratory symptoms, administer directly observed TB treatment and conduct child and adult TB contact tracing [12]. In other types of PHC units – hereafter referred to as “traditional” PHC clinics, nurse aids do much of this work, although they do not perform home visits. In summary, auxiliary health workers should have a crucial role in TB control in Brazil, including counselling and accruing contacts to attend nurse and medical visits in the PHC clinics.

Our group conducted a public health evaluation of the cascade-of-care for household contacts of TB cases in three Brazilian cities with the highest TB incidence rates in the country - Rio de Janeiro, Manaus and Recife [13] in order to implement solutions to increase uptake and treatment of LTBI, as part of a public health intervention study [14]. We found that less than 9% of identified contacts underwent investigation for LTBI and less than 3% started treatment. In order to understand the reasons for these findings, we conducted a knowledge, attitudes and practices (KAP) survey about *Mycobacterium tuberculosis* transmission and LTBI treatment among patients and contacts [13], nurses and physicians [15] and auxiliary healthcare workers in the three cities. We here describe the findings among this latter group.

## Method

### Study design

A KAP [16] survey was carried out from May 2015 to January 2016 among auxiliary healthcare workers.

### Setting

Although Brazil is a TB high-burden country, TB incidence rate is only 37/100,000 population [1]. Rio de Janeiro, Manaus and Recife have the highest TB incidence rates in the country, around 100/100,000 population [17]. The Brazilian public health system - the Unified Health System (SUS), is the world’s largest public health system and provides free-of-charge healthcare for all citizens as guaranteed by the Brazilian Constitution [18]. Through the past two decades, Brazil has been expanding access to PHC, concentrating efforts to educate, promote health and treat citizens, despite the great social disparities across the country. In the 90s, the Ministry of Health recommended the Family Health Strategy as the main PHC model [11]. Family health teams are composed of one physician, one nurse, one aid nurse and four to ten community health agents. The latter are individuals living in the clinic’s catchment area community, with “secondary” education level, corresponding to senior high school, who undergo basic training to perform surveillance and counselling tasks in critical health programs (immunization, pre-natal care, non-communicating chronic diseases, TB and leprosy among others). Each team is responsible for 1000 families (3000–4500 persons) living in the clinic’s catchment area [11]. By the time of data collection, the Family Health Strategy covered 46.2% of the population in Rio, 35.4% in Manaus and 53.9% in Recife [9]. The number of participating units (18) was proportional to the network installed in each municipality [9]. Eighteen units with the highest number of new TB cases were selected, representing traditional PHC, Family Health Clinics and reference PHC TB services in semirural and urban areas across the cities. Current guidelines from the National TB Program recommend investigation of all adult and child contacts with tuberculin skin testing (TST), plus chest X-rays for all contact children and for any contact with a positive TST or with symptoms before starting LTBI treatment [19]. Six to 9 months of isoniazid is the current recommended regimen for LTBI [19].

### Participants

Any auxiliary health worker (i.e., community health agents and nurse aids) working in the 18 clinics with the highest numbers of TB cases in the previous 12 months were eligible to voluntarily participate in the study. They were approached for consent according to their availability on the day of the visit to the clinics and were included after providing written informed consent. No information was available on variability of knowledge from previous studies in this population. A simple sample size calculation around a proportion with good knowledge on a particular question showed that at least 100 individuals would need to be recruited to maintain a 95% confidence interval of < 0.1 throughout a full range of proportions above 0.05. We set a target of at least 120 individuals, noting the number of questions and sites of recruitment increased the inherent variability in the study.

### Instrument

The English version of the questionnaire from a previous public health evaluation in Indonesia healthcare workers [20] (Addtional file 3), composed of 24 questions, was translated to Portuguese, then retro-translated to English for validation and reporting purposes (Additional file 2: Table S1). It was then adapted to the Brazilian context and to the targeted population (Additional file 2: Table S1 and Additional file 4). We suppressed questions that implicated in specific knowledge of diagnosis and treatment, leaving only questions that were compatible with the expected knowledge and tasks of auxiliary workers’ educational status. This was done by a panel constituted of one author (AT) and two independent faculty members with wide experience in TB care both in reference and in PHC facilities. We also suppressed questions that referred to payment of tests or drugs, since health care is free-of-charge in Brazilian public health facilities. Specific adaptations were also needed because of differences in the national guidelines. For example, in Indonesia, preventive therapy is only recommended for children under 5, regardless of TST while in Brazil, it is recommended for any TST-positive contact, regardless of age. Neither the Indonesian nor the Portuguese versions of this questionnaire were validated.

### Data collection

The questionnaire was applied orally by interviewers, who classified the oral response in pre-defined answers. They were health care personnel not involved in the selected facilities or with the analyses (see details below), hired specifically for this study. All interviewers were trained by the principal investigator before the start of data collection to read questions without reinforcement of replies, i.e., no “correct” answer was expected from the interviewee and to classify the respondents’ answers.

A pilot study was conducted with 10 non-target population (students) and 20 target population respondents in Rio de Janeiro (not included in the 135 respondents of the final version). The pilot tested comprehension by respondents and also aimed to list as many different answers as possible, which were successively added to the questionnaire’s answer options. Testing for reliability and consistency were not carried out, since this survey targeted a quick diagnosis to implement solutions; validation of the instrument was beyond our scope.

After the pilot, two questions were added because at the time of this survey, tuberculin was not available to all and a technical note [21] restricted use of tuberculin to specific high-risk populations. The final version (available as Additional files 1, 2, 3 and 4) included 23 open-ended questions on knowledge of TB symptoms, of TB transmission and prevention, perceptions on the importance of LTBI treatment, TB health care services and reasons for low adherence to contact investigation, as well as their practices regarding counselling.

### Data analysis

Although the answers were open, expected answers were objective and meant to be quantitatively analysed. More than one answer was admitted. Respondents spontaneously gave answers that were classified by the interviewer in defined categories (e.g: to prevent disease progression among contacts diagnosed with LTBI, responses such as using isoniazid regardless of dose or simply using “medication” were considered the same category, see Additional file 2: Table S2). During the pilot study, categories of answers were added as new answers were provided by interviewees and could not be classified in any of the pre-planned categories. Answers were further classified by the research team as satisfactory (not necessarily entirely correct) or not according to criteria set by the panel of three specialists, who defined if the answers were acceptable or not, i.e., if they would hamper or delay appropriate contact investigation and management, based on the same three specialist panel. At least one essential correct answer (marked as *, see Additional file 2: Table S2) was considered satisfactory provided that no unacceptable answer (marked as †) was given simultaneously, even if “neutral” answers were also given. For example, for a satisfactory answer of prevention of TB infection among index TB contacts, it was acceptable to answer “better nutrition” (neutral answer) as long as the respondent was also aware of air born transmission, i.e., answered “by not sleeping in the same room or bed”, or “ventilating the house” or “index cases using masks” provided they did not answer “by avoiding the same utensils/toothbrush” or “by BCG vaccination”, which could imply in inappropriate counselling).

The collected data were stored in Microsoft Office Excel (version 2010, Microsoft USA), where frequencies were generated.

## Results

One hundred and thirty-five auxiliary health workers - 96 community health workers and 39 nurse aids - were interviewed. The median interview duration was 13 [interquartile range (IQR) 10–18] minutes. They had been working with TB care for a median time of 47 (IQR 22–72) months; 89 (66%) had received TB training in the past 10 years, including 49 (36%) in the previous 12 months. The main topics of trainings are listed in Table 1. A minority had been trained on TST (18%) or TB prevention (27%).

**Table 1:** Tuberculosis training themes among 89 auxiliary healthcare workers interviewed in Rio de Janeiro, Manaus and Recife, Brazil. 2015–2016

Training themes	n (89)	%
Active TB patient counselling	37	42
Active TB patient care	50	56
Search for respiratory symptomatic persons	44	49
Treatment of active TB (directly observed therapy)	61	69
Contact identification	26	29
Cause of TB	32	36
TB prevention	26	29
How to collect sputum	33	37

Although 103 (76%) knew how to prevent person-to-person transmission, several gaps in knowledge were identified: 87 (64%) could not clearly distinguish LTBI from active tuberculosis; 85 (63%) did not know how to diagnose LTBI, 70 (52%) did not know how to prevent progression to active TB (Table 2 and Additional file 2: Table S2).

**Table 2:** Satisfactory answers about tuberculosis transmission and prevention from 135 auxiliary healthcare workers interviewed in Rio, Recife and Manaus, Brazil. 2015–2016

Questions	Satisfactory answers	National guidelines recommendations
Knowledge		
There is TB disease and TB infection (or latent TB). Do you know what the differences are?	48 (36%)	self-explaining
How can one say that the person is infected with the tuberculosis bacillus?	50 (37%)	positive TST or IGRA
How do you prevent that a household contact should be infected?	103 (76%)	cough hygiene, ventilated house
How do you prevent a person once infected from becoming ill?	65 (48%)	preventive isoniazid therapy (6–9 months)
According to the NTP, what intra-household contacts should receive treatment for TB prevention?	38 (28%)	all with positive TST/IGRA and normal chest X-ray
In the absence of available PPD, what intra-household contacts should receive treatment for TB prevention?	3 (2%)	HIV-infected individuals and children (< 16)
Attitudes/Perceptions		
Do you think it is important for a child who lives with a patient with active TB to be screened for active TB?	134 (99%)	yes
Do you think it is important for a child who lives with a patient with active TB to be screened for latent TB?	129 (96%)	yes
Do you think it is important for an adult living with an active TB patient to be screened for active TB?	131 (97%)	yes
Do you think it is important for an adult living with an active TB patient to be screened for latent TB?	133 (99%)	yes
Do you think the health unit you work in should be responsible for investigating contacts who live with a patient with active TB, or should they do it elsewhere?	103 (76%)	depends on the clinic
What are the difficulties of this clinic to evaluate a contact that lives with a patient with TB disease?	92 (68%)	depends on the clinic
Sometimes parents / guardians may not bring them to the investigation. When that happens, what do you think are the main reasons?	97 (72%)	see Additional file 2: Table S2
Sometimes adult contacts do not come to the unit to be investigated. Which do you think are the main reasons?	114 (84%)	see Additional file 2: Table S2
Practices		
What do you do for an adult, contact of a patient living in the same household who had a recent TB diagnosis?	96 (71%)	refer to investigation
What do you do for a child, contact of a patient living in the same household who had a recent TB diagnosis?	109 (81%)	refer to investigation
What do you do if a child using isoniazid for the treatment of latent TB has nausea?	128 (95%)	change timing of medication uptake and refer to the nurse or doctor
What do you do if adult using isoniazid for the treatment of latent TB has nausea?	135 (100%)	
What do you do if a child using isoniazid for the treatment of latent TB goes yellow?	127 (94%)	suspend medication and refer to the nurse or doctor
What do you do if a adult using isoniazid for the treatment of latent TB goes yellow?	126 (93%)	

*TB* Tuberculosis, *LTBI* Latent tuberculosis infection, *NTP* National TB Program

Most believed that it is important to investigate adult (99%) and child (96%) contacts for LTBI, although perceived barriers to do so were unsound: 11 (8%) do not perceive contact identification and referral as part of their attributed work, 11 (8%) reported being too busy, and 7 (5%) not having adequate training (Table 2 and Additional file 2: Table S2). When asked why contacts do not show up, reasons were mostly, according to their perceptions, attributed to index cases and contacts: 65 (48%) answered that parents do not understand the importance of this investigation for their children; 59 (44%) that adults do not understand the importance of this investigation for themselves and 45 (33%) the TB cases and contacts fear stigma (Additional file 2: Table S2).

In relation to practices, most recognized that they do not invite contacts to the clinic [123 (91%) for children and 71 (53%) for adults], although only 34 (25%) perceived difficulties related to investigation in their clinic (Table 2). Practices related to contact treatment (management of adverse events) were satisfactory (Table 2).

There were no significant differences between job categories (nurse aids versus community health agents) or among the three cities.

## Discussion

Many losses to the cascade-of-care of TB contacts have been shown [4]. In Brazil, most losses occur in the first steps: contact identification and referral for investigation [13]. Although treatment of adult and child contacts with LTBI has been recommended since 2010 in the country less than 9% of contacts are even identified by health services [13]. We aimed to understand the reasons for these high losses in the initial steps of the cascade-of-care of contacts, by interviewing relevant players in this process [13, 15]. In the current study, we present the results among auxiliary healthcare workers, key actors in case contact management in many LMIC [22, 23]. We found that there were relevant gaps in knowledge and practices – mainly those regarding their key duty: to identify and invite contacts for investigation and eventual treatment in the health clinic.

Auxiliary health care providers, especially community health workers, have fulfilled PHC needs in LMIC, where there is shortage of clinical workforce [8]. More recently, with the aging population and the increasing costs of health in high-income countries, community health agents have been closing health gaps and reducing disparities in access to universal care, particularly among elders [24]. Education of the community is among their tasks and its efficacy has been a game changer in infectious disease outbreaks [25]. However, building this kind of workforce is challenging. Gaps in KAP among auxiliary health workers have been reported in other areas, such as obstetrics [26–28] and other infectious diseases [29–31]. To the best of our knowledge, this is the first KAP survey on TB transmission and prevention among auxiliary health care workers, and relevant gaps were also found.

Knowledge gaps were notably related to LTBI management, including how to recognize it and prevent progression to active TB through treatment. These gaps were expected since those topics are seldom included in their training and they do not acquire field experience. The great majority had satisfactory attitudes, i.e., they thought it is important to investigate contacts. Contrasting with the recognition of the importance of LTBI and active TB investigation, a relevant finding worth exploring by TB managers was that most auxiliary workers do not invite contacts for nurse/physician visits in the clinics, as recommended by the National Guidelines [19]. They mainly attributed this to TB service users: index cases and contacts fear stigma, contacts are too busy and would not come anyway. However, this was not what our group found when interviewing users in the same cities and period. Other reasons include difficulties for investigation in the clinics but all clinics do offer TST and X-rays are available in the clinic itself, with the exception of one, which refers contacts to a nearby facility.

Despite these gaps, practices regarding adverse events were very adequate, probably because they are trained and experienced in dealing with adverse events of the active TB regimen, which also contains isoniazid.

In December 2016, the Brazilian congress approved an amendment to the Constitution to reduce public spending [32]. The catastrophic consequences on health were evident 2 years later [33]. A later regulation [34] established funds to be invested in primary care health teams, including financial incentives to healthcare workers. Unfortunately, community health agents are not entitled to receive these incentives and their training and support is not even mentioned in the document. Without investments of health systems in capacity building and support of auxiliary workers, strengthening of primary care quality are unlikely.

Our study has a few limitations. The questionnaire was applied by interviewers. This may have created some embarrassment although interviewees were reassured that answers would be kept anonymous for analyses, and interviewers were independent from the health service. Although interviewers were extensively trained, misclassification of answers cannot be discarded. However, answers were very objective and any uncertainty was clarified by the principal investigator in Brazil (AT). Because the instrument was not validated, reproducibility and consistency were not evaluated. In addition, attitudes regarding TB prevention may be difficult to capture, especially with the questions as posed (“Do you think it is important …” ), and could explain the contradiction between attitudes and practices. A Likert scale-based answer might have been more useful, and we would recommend it for further research. Finally, only three cities were investigated, which limits the generalizability of our findings in a very large country. However, we tried to include different kinds of healthcare services, from the least specialized (Family Health Clinics) to Aids/TB reference clinics, all available in the primary care network.

A recent individual-based data bank analysis has shown that TB patients covered by the Family Health Service in Rio de Janeiro are more likely to have successful TB outcomes than those not covered [10]. The efficacy of their actions are well shown in prevention and management of other diseases [35]. We believe that, if appropriately trained and above all sensitized to this relevant task, auxiliary healthcare workers could play an important role in the improvement of TB prevention. Of course, teamwork is necessary to ensure that contacts are not lost during investigation and over the course of the treatment when indicated. As part of the solutions implemented in six of these clinics, we trained healthcare workers, including community health agents, with encouraging preliminary results, i.e. substantial decrease in losses of all steps of the cascade [36].

## Conclusions

Auxiliary healthcare workers in Brazil have important knowledge gaps despite their perception of the importance of TB prevention among contacts. They need to incorporate contact referral as one of their tasks to enable progress toward the target of TB elimination. At a supra-national level, international institutions such as the World Health Organization should advocate for international recognition of mid-level providers in order for them to acquire the necessary legitimacy in the health system.

## Additional files

Additional file 1: Raw de-identified dataset analysed in this study, in Microsoft® format. (XLSX 152 kb)

Additional file 2: **Table S1.** Retro-translated questionnaire and **Table S2.** Details on interviewees answers to the questionnaire. (DOCX 131 kb)

Additional file 3: The original questionnaire published by Hill et al, on which our questionnaire was based. (DOC 60 kb)

Additional file 4: The Brazilian version of the questionnaire, adapted from Hill et al. (DOC 94 kb)

## Data Availability

All data generated or analysed during this study is available upon reasonable demand to the corresponding author.

## Abbreviations

IQR: Interquartile range
KAP: Knowledge, attitudes and practices
LMIC: Low and medium-income countries
LTBI: Latent tuberculosis infection
NTP: National TB Program
PHC: Primary health care
SUS: Unified Health System (the Brazilian public health system)
TB: Tuberculosis
TST: Tuberculin skin testing
