# Supplementary material for: Knowledge, attitudes and practices on tuberculosis transmission and prevention among auxiliary healthcare professionals in three Brazilian high-burden cities: a cross-sectional survey
Source: BMC Health Serv Res. 2019 Jul 30;19:532. doi: 10.1186/s12913-019-4231-x (PMC6668184; doi:10.1186/s12913-019-4231-x)
Supplement: Supplementary file 2 — Table S1. Retro-translated questionnaire and Table S2. Details on interviewees answers to the questionnaire. (DOCX 131 kb) [file 12913_2019_4231_MOESM2_ESM.docx]

| **Table S1– Retro-translated Brazilian and Rutherford’s versions of the questionnaire (differences in bold)** | | |
| --- | --- | --- |
| **Databank**  **Question number** | **Brazilian questionnaire** | **Rutherford’s questionnaire** |
| 9a | **In the absence of available PPD, what intra-household contacts should receive treatment for TB prevention?** | According to the NTP, who should be given isoniazid as a TB prevention measure in Indonesia? **There was a shortage of tuberculin at the time of the study.** |
|  | **Supressed. Not related to LTBI.** | Which household contacts should be given immunization against TB? |
|  | **Supressed. Not a CHA assignment.** | What are the current guidelines for isoniazid prophylactic therapy in children? |
|  | **Supressed. Not a CHA assignment.** | What is the minimum time period isoniazid prophylactic therapy should be taken? |
|  | *Attitudes* | *Attitudes* |
| 10 | Do you think it is important for a child who lives with a patient with active TB to be screened for active TB? | **What, if anything makes you worried about checking children who live with a TB case for TB disease.** |
| 11 | Do you think it is important for a child who lives with a patient with active TB to be screened for latent TB? | **Do you think that children <5yrs who live with a TB case and do not have TB disease should be given isoniazid prophylactic therapy?** |
| 12 | Do you think it is important for an adult living with an active TB patient to be screened for active TB? | **This was added because Brazilian guidelines recommend investigation and treatment of LTBI in adults as well, unlike Indonesian guidelines** |
| 13 | Do you think it is important for an adult living with an active TB patient to be screened for latent TB? | **This was added because Brazilian guidelines recommend investigation and treatment of LTBI in adults as well, unlike Indonesian guidelines** |
| 14 | Do you think the health unit you work in should be responsible for investigating contacts who live with a patient with active TB, or should they do it elsewhere? | Same question |
| 15 | What are the difficulties of this clinic to evaluate a contact that lives with a patient with TB disease? | **Added. We wanted to understand bottlenecks in order to implement local solutions in the future.** |
| 16 | Sometimes parents / guardians may not bring them to the investigation. When that happens, what do you think are the main reasons? | Same question |

| **Table S1 (continue) – Retro-translated Brazilian and Rutherford’s versions of the questionnaire (differences in bold)** | | |  |
| --- | --- | --- | --- |
| **Databank Question number** | **Brazilian questionnaire** | **Rutherford’s questionnaire** |  |
|  | *Practice* | *Practice* |  |
| 17 | Sometimes adult contacts do not come to the unit to be investigated. Which do you think are the main reasons? | **This was added because Brazilian guidelines recommend investigation and treatment of LTBI in adults as well, unlike Indonesian guidelines.** |  |
| 18 | What do you do for an adult, contact of a patient living in the same household who had a recent TB diagnosis? | **This was added because Brazilian guidelines recommend investigation and treatment of LTBI in adults as well, unlike Indonesian guidelines.** |  |
| 19 | **What do you do for a child, contact of a patient living in the same household who had a recent TB diagnosis? (They could give information or forward to the clinic to have more information.)** | When you see an adult TB patient what of the following information do you tell them? |  |
| 20 | What do you do if a child using isoniazid for the treatment of latent TB has **nausea**? | What would you do if a child on prophylactic therapy presented with minor side effects? **We preferred to give a concrete example.** |  |
| 21 | What do you do if adult using isoniazid for the treatment of latent TB has **nausea**? | **This was added because Brazilian guidelines recommend investigation and treatment of LTBI in adults as well, unlike Indonesian guidelines.** |  |
| 22 | What do you do if a child using isoniazid for the treatment of latent TB **goes yellow**? | What would you do if a child on prophylactic therapy presented with major side effects? **We preferred to give a concrete example.** |  |
| 23 | What do you do if a adult using isoniazid for the treatment of latent TB **goes yellow**? | **This was added because Brazilian guidelines recommend investigation and treatment of LTBI in adults as well, unlike Indonesian guidelines.** |  |

CHA=community health agent

**Table S2- Details on answers about knowledge, attitudes and practice regarding tuberculosis transmission and prevention among 135 auxiliary health workers interviewed in Rio de Janeiro, Manaus and Recife, Brazil. 2015-2016**

| **Questions and answers** | **n (135)** | **%** |
| --- | --- | --- |
| **What is difference between active TB and LTBI?** |  |  |
| Does not know the difference † | 65 | 48 |
| The person with TB has cough * | 6 | 4 |
| The person with TB has symptoms and contacts with LTBI do not* | 29 | 21 |
| The person with TB has fever * | 3 | 2 |
| The person with TB loses weight * | 4 | 3 |
| The person with latent TB has no symptoms * | 27 | 20 |
| Other | 4 | 3 |
| Satisfactory | **48** | **36** |
| **How can one affirm that the person is infected with the tuberculosis bacillus?** |  |  |
| Person asymptomatic with pulmonary tuberculosis | 0 | 0 |
| Person asymptomatic with extrapulmonary tuberculosis | 10 | 7 |
| Person with positive tuberculin skin test (“PPD”) or IGRA * | 37 | 27 |
| Asymptomatic person | 24 | 18 |
| Person with normal chest x-ray | 23 | 17 |
| Person with positive tuberculin skin test (PPD) or IGRA and normal chest X-ray * | 8 | 6 |
| Asymptomatic person with positive tuberculin skin test (PPD) or IGRA and normal chest X-ray * | 15 | 11 |
| Person with positive AFB (or sputum or sputum smear) test † | 49 | 36 |
| I do not know † | 18 | 13 |
| Other | 14 | 10 |
| Satisfactory | **50** | **37** |
| **How can a contact (person who lives with a patient with a recent diagnosis of active TB) be prevented from becoming infected with TB?** |  |  |
| Sleeping in a bed different from that of the TB patient * | 10 | 7 |
| Do not get too close to the index case * | 29 | 21 |
| Index case should wear mask or handkerchief when coughing * | 74 | 55 |
| Resting, not making effort | 5 | 4 |
| Not sharing utensils with the patient with TB † | 15 | 11 |
| Not sharing their toothbrush with the patient with TB † | 7 | 5 |
| Quitting smoking | 4 | 3 |
| Stopping consuming alcohol | 6 | 4 |
| Better nutrition | 21 | 16 |
| Taking the BCG vaccine † | 4 | 3 |
| Taking isoniazid or other treatment for latent infection (“chemoprophylaxis”) | 23 | 17 |
| Keep the house ventilated * | 70 | 52 |

* At least one option quoted to consider answer as satisfactory

† No options cited to consider answer as satisfactory

FHC = family health clinic, LTBI = latent tuberculosis infection, INH = isoniazid, TB = tuberculosis

**Table S2 (continue)- Details on answers about knowledge, attitudes and practice regarding tuberculosis transmission and prevention among 135 auxiliary health workers interviewed in Rio de Janeiro, Manaus and Recife, Brazil. 2015-2016**

| **Questions and answers** | **n (135)** | **%** |
| --- | --- | --- |
| Take vitamins | 2 | 1 |
| Do not know † | 4 | 3 |
| Medical monitoring | 10 | 7 |
| Others | 18 | 13 |
| Satisfactory | **103** | **76** |
| **How do you prevent a person - once infected - from becoming ill with TB?** |  |  |
| Sleeping in a bed different from that of the TB patient * | 1 | 1 |
| Do not get too close to the index case | 6 | 4 |
| Should index case wear mask or handkerchief when coughing | 30 | 22 |
| Rest and not make an effort | 5 | 4 |
| Do not share utensils with the patient with TB † | 4 | 3 |
| Do not share toothbrush with the patient with TB † | 3 | 2 |
| Quitting smoking | 12 | 9 |
| Stopping consuming alcohol | 11 | 8 |
| Eating better | 34 | 25 |
| Taking the BCG vaccine † | 7 | 5 |
| Taking isoniazid or other treatment for latent infection (chemoprophylaxis) * | 37 | 27 |
| Keep the house ventilated | 33 | 24 |
| Take vitamins daily | 3 | 2 |
| With medicines (does not know the name) * | 30 | 22 |
| Weekly medical follow-up | 7 | 5 |
| Do not know† | 14 | 10 |
| Others | 30 | 22 |
| Satisfactory | **65** | **48** |
| **According to PNCT recommendation, what intra-household contacts should receive treatment for TB prevention? (Guidelines previous to 2010 recommended treatment for children under the age of 15 (not adults)** |  |  |
| All * | 31 | 23 |
| All, as long as active TB is ruled out * | 18 | 13 |
| All without active TB and with a positive PPD or IGRA * | 21 | 16 |
| All children under the age of 15 † | 3 | 2 |
| All children under the age of 15, provided that active TB is ruled out † | 0 | 0 |
| All children under the age of 15, without active TB and with a positive PPD or QFT † | 4 | 3 |
| All children under the age of 5 † | 4 | 3 |
| All children under the age of 5, provided that active TB is ruled out † | 2 | 1 |
| All children under the age of 5, without active TB and with a positive PPD or QFT † | 1 | 1 |

* At least one option quoted to consider answer as satisfactory

† No options cited to consider answer as satisfactory

FHC = family health clinic, LTBI = latent tuberculosis infection, INH = isoniazid, TB = tuberculosis

**Table S2 (continue)- Details on answers about knowledge, attitudes and practice regarding tuberculosis transmission and prevention among 135 auxiliary health workers interviewed in Rio de Janeiro, Manaus and Recife, Brazil. 2015-2016**

| **Questions and answers** | **n (135)** | **%** | |
| --- | --- | --- | --- |
| Prevention is not recommended in Brazil† | 2 | 1 | |
| I do not know† | 44 | 33 | |
| Satisfactory | **38** | **28** | |
| **According to the current PNCT recommendations, in the absence of available PPD, what intra-household contacts should receive treatment for TB prevention? (There was a shortage of PPD during the period of the study, because of discontinuation of manufacture by the supplier)** |  |  | |
| All * | 45 | 33 | |
| All, as long as active TB is ruled out * | 25 | 19 | |
| All children under 15 years old † | 9 | 7 | |
| Everyone under 15 years of age, provided that active TB is removed † | 3 | 2 | |
| All children under 5 years of age, provided that active TB is removed † | 0 | 0 | |
| Under 5 years old, without active TB † | 3 | 2 | |
| Prevention is not recommended in Brazil † | 5 | 4 | |
| I do not know † | 37 | 27 | |
| Others | 32 | 24 | |
| Satisfactory | **3** | **2** | |
| **Do you think it is important for a child who lives with a patient with active TB to be screened for active TB?** |  |  | |
| Yes = Satisfactory | **134** | **99** | |
| **Do you think it is important for a child who lives with a patient with active TB to be screened for latent TB**? |  |  | |
| Yes = Satisfactory | **129** | **96** | |
| **Do you think it is important for an adult living with a patient with active TB to be screened for active TB?** |  |  | |
| Yes = Satisfactory | **131** | **97** | |
| **Do you think it is important for an adult living with an active TB patient to be screened for latent TB?** |  |  | |
| Yes = Satisfactory | **133** | **99** | |
| **Do you think that the health unit in which you work should be responsible for investigating contacts who live with a patient with active TB, or should they do it in another location?** |  |  | |
| This health facility should investigate children but not adults | 0 | 0 | |
| This health facility should investigate adults, but not children | 22 | 16 | |
| This health facility should investigate adults and children * | 103 | 76 | |
| This health facility should not investigate adults or children, but refer them elsewhere | 11 | 8 | |
| It is not necessary to investigate them † | 0 | 0 | |
| Others | 12 | 9 | |
| Satisfactory | **103** | **76** | |
| **What are the difficulties of this clinic to evaluate a contact that lives with a patient with active TB?** |  |  |  |
| We have no difficulty investigating contacts here* | 36 | 27 |  |
| We are too busy in our health unit † | 11 | 8 |  |
| I was not trained properly to evaluate contacts | 7 | 5 |  |

* At least one option quoted to consider answer as satisfactory

† No options cited to consider answer as satisfactory

FHC = family health clinic, LTBI = latent tuberculosis infection, INH = isoniazid, TB = tuberculosis**Table S2 (continue) - Details on answers about knowledge, attitudes and practice regarding tuberculosis transmission and prevention among 135 auxiliary health workers interviewed in Rio de Janeiro, Manaus and Recife, Brazil. 2015-2016**

| **Questions and answers** | **n (135)** | **%** |
| --- | --- | --- |
| We fear resistance to medication† | 0 | 0 |
| Methods for TB investigation in this health facility are not good | 2 | 1 |
| We do not have X-ray | 2 | 1 |
| This is not a priority † | 0 | 0 |
| I do not believe that the treatment reduces the risk of getting ill with TB | 0 | 0 |
| I'm afraid of the side effects † | 1 | 1 |
| The extra workload stresses me † | 2 | 1 |
| I do not feel secure when evaluating contacts for active TB | 2 | 1 |
| All children are vaccinated with BCG and do not need other preventive measures | 1 | 1 |
| I do not have training to do PPD | 4 | 3 |
| The contacts do not show up in the health unit | 38 | 28 |
| We do not have PPD | 28 | 21 |
| Sputum samples delivery to labs is delayed | 8 | 6 |
| Others | 45 | 33 |
| Satisfactory | **92** | **68** |
| **Sometimes the parents / guardians of children may not bring them to the investigation. When that happens, what do you think are the main reasons?** |  |  |
| They always bring | 32 | 24 |
| They do not understand how important the investigation of their children is | 65 | 48 |
| They cannot afford to bring their children to be investigated | 7 | 5 |
| They only come to the health unit when the children are sick | 19 | 14 |
| They prefer to take the children to be investigated in another health unit | 1 | 1 |
| They are too lazy to take the children to be investigated † | 15 | 11 |
| They are not meant to bring the children to be investigated | 8 | 6 |
| They do not show up because of fear of stigma † | 13 | 10 |
| They have believe in mystical treatment † | 4 | 3 |
| The index cases are not able to bring their children because they are also ill | 0 | 0 |
| I do not know † | 8 | 6 |
| Others | 28 | 21 |
| Satisfactory | **97** | **72** |
| **Sometimes adult contacts do not come to the unit to be investigated. Which do you think are the main reasons**? |  |  |
| They always come † | 31 | 23 |
| They do not understand how important this investigation is | 59 | 44 |
| They cannot afford to come to the health facility | 4 | 3 |
| Adults only come to the clinic when they are sick | 43 | 32 |
| They go to other units or hospitals to be investigated | 2 | 1 |
| They are too lazy to come to the unit to be investigated † | 3 | 2 |
| Adults are not advised to come to the health facility for investigation | 11 | 8 |
| Adults are afraid to come because of fear of stigma † | 45 | 33 |

* At least one option quoted to consider answer as satisfactory

† No options cited to consider answer as satisfactory

FHC = family health clinic, LTBI = latent tuberculosis infection, INH = isoniazid, TB = tuberculosis

**Table S2 (continue) - Details on answers about knowledge, attitudes and practice regarding tuberculosis transmission and prevention among 135 auxiliary health workers interviewed in Rio de Janeiro, Manaus and Recife, Brazil. 2015-2016**

| **Questions and answers** | n (135) | % |
| --- | --- | --- |
| They do not have time to come to the unit, they need to work | 13 | 10 |
| I do not know † | 4 | 3 |
| Others | 20 | 15 |
| Satisfactory | **114** | **84** |
| **What do you do with an adult who lives with a newly diagnosed patient with tuberculosis?** |  |  |
| I refer them to a doctor or a nurse in the clinic * | 96 | 71 |
| I ask if they have any symptoms | 66 | 49 |
| I refer them for a TST | 22 | 16 |
| I recommend sputum examination | 35 | 26 |
| I do not do anything † | 0 | 0 |
| I do not know † | 2 | 1 |
| Others | 32 | 24 |
| Satisfactor**y** | **96** | **71** |
| **What do you do for a child, contact of a patient living in the same household who had a recent TB diagnosis?** |  |  |
| I refer them to a doctor or a nurse in the clinic * | 109 | 81 |
| I ask if you have any symptoms | 50 | 37 |
| Forward to make PPD | 10 | 7 |
| I recommend sputum examination | 12 | 9 |
| I do not do anything† | 0 | 0 |
| I do not know † | 4 | 3 |
| Others | 31 | 23 |
| Satisfactory | **109** | **81** |
| **What do you do if a child using isoniazid for the treatment of latent TB has any side effect to the nausea-type medication?** |  |  |
| I refer to a doctor or nurse in the clinic * | 110 | 81 |
| I refer to a specialized unit | 7 | 5 |
| I recommend to insist on treatment * | 46 | 34 |
| I recommend to stop the treatment † | 4 | 3 |
| I do not know † | 3 | 2 |
| Others | 13 | 10 |
| Satisfactory | **128** | **95** |
| **What do you do for an adult in treatment for latent TB with isoniazid that has any side effects such as nausea**? |  |  |
| I refer to a doctor or nurse in the clinic * | 119 | 88 |
| I recommend to insist on treatment * | 51 | 38 |
| I recommend to stop the treatment † | 0 | 0 |
| I do not know † | 0 | 0 |

* At least one option quoted to consider answer as satisfactory

† No options cited to consider answer as satisfactory

FHC = family health clinic, LTBI = latent tuberculosis infection, NH = isoniazid, TB = tuberculosis

**Table S2 (continue) – Details on answers about knowledge, attitudes and practice regarding tuberculosis transmission and prevention among 135 auxilairy health workers interviewed in Rio de Janeiro, Manaus and Recife, Brazil. 2015-2016**

| **Questions and answers** | n (135) | % |
| --- | --- | --- |
| Others | 9 | 7 |
| Satisfactory | 135 | 100 |

| **What do you do if a child using isoniazid for the treatment of latent TB becomes yellow?** |  |  |
| --- | --- | --- |
| I refer to a doctor or nurse in the clinic * | 116 | 86 |
| Forward to a specialized unit | 119 | 88 |
| I recommend to insist on treatment † | 8 | 6 |
| I recommend to stop the treatment * | 10 | 7 |
| I do not know † | 9 | 7 |
| Others | 4 | 3 |
| Satisfactory | 127 | 94 |
| What do you do if an adult using isoniazid for the treatment of latent TB becomes yellow? |  |  |
| I refer to a doctor or nurse in the clinic * | 119 | 88 |
| I recommend to insist on treatment † | 9 | 7 |
| I recommend to stop the treatment * | 13 | 10 |
| I do not know | 10 | 7 |
| Others | 4 | 3 |
| Satisfactory | 126 | 93 |

* At least one option quoted to consider answer as satisfactory

† No options cited to consider answer as satisfactory

FHC = family health clinic, LTBI = latent tuberculosis infection, NH = isoniazid, TB = tuberculosis

|  |  |  |
| --- | --- | --- |
