# Supplementary material for: Knowledge, attitudes and practices on tuberculosis transmission and prevention among auxiliary healthcare professionals in three Brazilian high-burden cities: a cross-sectional survey
Source: BMC Health Serv Res. 2019 Jul 30;19:532. doi: 10.1186/s12913-019-4231-x (PMC6668184; doi:10.1186/s12913-019-4231-x)
Supplement: Supplementary file 3 — The original questionnaire published by Hill et al, on which our questionnaire was based. (DOC 60 kb) [file 12913_2019_4231_MOESM3_ESM.doc]

| Staff member ID number (*for researcher to complete*) |__|__|__|__| | |
| --- | --- |
| Date of interview (*for researcher to complete*) |__|__|/|__|__|/|__|__|  Day / Month / Year | |
| We would like to ask you a few questions about your training, your involvement in TB patient and case contact management and your knowledge about latent TB infection and isoniazid prophylactic therapy. We would also like to ask your opinion of managing children who live with a TB case. This is not a test; there are no right or wrong answers. We realise how limited your time is and greatly appreciate your taking the time to complete this questionnaire. We expect it will take no more than 10-20minutes. | |
| **Questionnaire 03: Health Care Worker Assessment** | |
| 1. | What is your position at health centre?  |__| Doctor  |__| Nurse  |__| Educator/Counsellor  |__| Specialist (*please specify*)___________________________  |__| Other (*please specify*)______________________________ |
| 2. | How long have you been an employee at health centre? |__|__| years |
| 3. | Have you had training in the past year?  |__| Yes  |__| No (*please go to question 5*) |
| 4. | If yes, what kind of training did you have? *(tick as many boxes as apply)*  |__| Training in basic patient counselling  |__| Training in patient management  |__| Training in paediatric TB  |__| Training in TB diagnostics  |__| Training in TB medications (types, dosages etc.)  |__| Training in case contact management  |__| Training in VCT counselling  |__| Other (*please specify)_______________________________________* |
| 5. | When you see an adult TB patient what of the following information do you tell them? *(tick as many boxes as apply)*  |__| How to take their medication *(i.e. time of day, with food etc.)*  |__| How long to take their medication for  |__| When to come back to the clinic  |__| How to protect other people in their family from becoming infected  |__| What to do if side effects occur  |__| To bring all the people they live with to the clinic to be checked  |__| To bring only their children to the clinic to be checked  |__| To stop smoking *(if relevant)*  |__| Nutritional information  |__| Not applicable to my position |

| 6. | What of the following options can stop someone living with a TB case from becoming sick with TB themselves? *(tick as many boxes as apply)*  |__| = Sleeping in a different bed from the TB case  |__| = Resting and not doing hard work  |__| = Not sharing eating utensils with the TB case  |__| = Not sharing a toothbrush with the TB case  |__| = Stopping smoking  |__| = Eating a better diet  |__| = Isoniazid or other prophylactic therapy  |__| = Keeping the house ventilated  |__| = Taking daily vitamins |
| --- | --- |
| 7. | Which definition do you think best describes a person with latent TB infection? (*please choose one option only*)  |__| The person is healthy but has *Mycobacterium tuberculosis* in their body  |__| The person is Mantoux positive, chest x-ray positive but has no symptoms  |__| The person has symptoms, a positive chest x-ray but is sputum smear negative |
| 8. | How would you diagnose latent TB infection? (*please choose one option only*)  |__| Do a fine needle aspirate on the cervical lymph nodes  |__| Do a Mantoux test  |__| Do a chest x-ray  |__| Take a sputum sample for ZN microscopy and culture  |__| Do a gastric aspirate  |__| Other, specify: ___________________  |__| I don’t know |
| 9. | Would you ever treat latent TB infection?  |__| Yes  |__| No |
| 10. | What things would you do first if a child (<5yrs) who lives with a TB case comes to be checked? (*please mark all clinical and diagnostic evaluations you would do as a first step)*  |__| Refer the patient to a specialist  |__| Do a symptom check  |__| Do a Mantoux test  |__| Do a chest x-ray  |__| Do a sputum smear test  |__| Culture sputum  |__| I would not conduct any tests  |__| I don’t know  |__| Other, specify: ___________________ |

| 11. | What would you give a young child (<5yrs) who lives with a TB case and does not have TB disease? (*please mark only one category*)  |__| If Mantoux positive give prophylactic therapy  |__| Give prophylactic therapy no matter what the Mantoux result  |__| Give full anti-TB therapy because child may be misdiagnosed  |__| Give BCG vaccination if Mantoux negative  |__| Give 7 days antibiotics  |__| Give vitamins  |__| Give advice to the parents but nothing to the child  |__| Other, specify: ___________________ |
| --- | --- |
| 12. | Can you please explain the policy to mange children (<5yrs) living with a TB case in your own words (*please choose one option only*)  |__| Screen child for TB disease. If disease free and Mantoux positive then give prophylactic therapy  |__| Screen child for TB disease. If disease free give prophylactic therapy  |__| Screen child for TB disease. If disease free do nothing more  |__| Give child antibiotics for 7 days to protect against TB infection  |__| Screen child using scoring system if 6 or more points place on full anti-TB medication.  |__| Give child vitamins and nutritional advice to their parents  |__| Tell parents to look for symptoms of TB  |__| I don’t know |
| 13. | According to the NTP, who should be given isoniazid as a TB prevention measure in Indonesia? *(tick as many boxes as apply)*  |__| All people living in the household of a TB case who don’t have TB disease  |__| All children (<15yrs) living in the household of a TB case who don’t have TB disease **AND** who are Mantoux positive  |__| All children (<5yrs) living in the household of a TB case who don’t have TB disease **AND** who are Mantoux positive  |__| All children (<5yrs) living in the household of a TB case who don’t have TB disease  |__| No one, we don’t do isoniazid therapy in Indonesia  |__| I don’t know |
| 14. | Which household contacts should be given immunization against TB? (*please choose one option only*)  |__| Only the children <5yrs  |__| Any household contact who is Mantoux negative and does not have TB disease  |__| I don’t know |
| 15. | What are the current guidelines for isoniazid prophylactic therapy in children? (*please tick the correct box)*  |__| = 2mg/kg/day  |__| = 5mg/kg/day  |__| = 10mg/ kg/day  |__| = 12mg/kg/day  |__| = I don’t know |

| 16. | What is the minimum time period isoniazid prophylactic therapy should be taken?  |__|__| Months  |__| I don’t know |
| --- | --- |
| 17. | Please mark the most common side effects from isoniazid prophylactic therapy. (*please mark as many options as apply)*  |__| Rash |__| Mood changes  |__| Nausea |__| Stomach pain  |__| Itchiness |__| Numbness  |__| Diarrhoea |__| Headache  |__| Heart burn |__| Cramps  |__| Hepatotoxicity |__| Vision changes  |__| Easy bruising and bleeding |__| Seizures  |__| Painful joint swelling |
| 18. | What would you do if a child on prophylactic therapy presented with minor side effects? (*please choose one option only*)  |__| Stop therapy  |__| Provide medication to alleviate side effects  |__| Continue therapy but monitor the child more closely  |__| Stop therapy until side effects have gone then continue  |__| Prescribe an alternative prophylactic therapy (i.e. rifampicin)  |__| I don’t know |
| 19. | What would you do if a child on prophylactic therapy presented with major side effects? (*please choose one option only*)  |__| Stop therapy  |__| Provide medication to alleviate side effects  |__| Continue therapy but monitor the child more closely  |__| Stop therapy until side effects have gone then continue  |__| Prescribe an alternative prophylactic therapy (i.e. rifampicin)  |__| I don’t know |
| In this next section we are interested in your opinion only. There are no right or wrong answers and we will not share your opinion with any of your colleagues. | |
| 20. | Do you think that children <5 years of age who live with a TB case should be checked for TB disease?  |__| Yes  |__| No |
| 21. | Do you think that the clinic you work at should be responsible for checking children who live with a TB case for TB disease, or should they be checked somewhere else? (*please choose one option only that best represents your opinion*)  |__| Checked here  |__| Checked somewhere else  |__| No need to check |

| 22. | Sometimes caregivers of children don’t bring their children to be checked. Why do you think this is? *(please tick as many boxes as apply)*  |__| Caregivers do not understand how important checking their children is  |__| Caregivers cannot afford to bring their children to the clinic for checking  |__| Caregivers only come to the clinic if their children are sick  |__| Caregivers would rather go to the PKM to have their children checked  |__| Caregivers are too lazy to have their children checked  |__| Caregivers are not told to come to the clinic to have their children checked  |__| I don’t know |
| --- | --- |
| 23. | Do you think that children <5yrs who live with a TB case and do not have TB disease should be given isoniazid prophylactic therapy?  |__| Yes  |__| No  |__| I don’t know |
| 24. | What, if anything makes you worried about checking children who live with a TB case for TB disease (*please tick as many boxes as apply*)  |__| We are too busy at the clinic already  |__| I don’t have the correct training to check children  |__| We could cause MDR-TB if we treat children with TB disease with preventive therapy only  |__| I don’t think the screening methods are very good at our clinic  |__| The children won’t take the preventive therapy even if I prescribe it  |__| The extra work load makes me feel stressed at work  |__| I do not feel confident when checking children for TB disease |
| That is the end of our questionnaire. Thank you for taking the time to complete this. | |
