# Supplementary material for: Knowledge, attitudes and practices on tuberculosis transmission and prevention among auxiliary healthcare professionals in three Brazilian high-burden cities: a cross-sectional survey
Source: BMC Health Serv Res. 2019 Jul 30;19:532. doi: 10.1186/s12913-019-4231-x (PMC6668184; doi:10.1186/s12913-019-4231-x)
Supplement: Supplementary file 4 — The Brazilian version of the questionnaire, adapted from Hill et al. (DOC 94 kb) [file 12913_2019_4231_MOESM4_ESM.doc]

| Numero de identificação do profissional |__|__|__|__| | |
| --- | --- |
| Nome da unidade de saúde __________________________________________ | |
| Data da entrevista |__|__|/|__|__|/|__|__| Hora do início:|__|__|:|__|__|  Dia / Mês / Ano | |
| *Neste questionário você encontrará algumas perguntas sobre seu conhecimento, experiência e envolvimento com pacientes com tuberculose (TB) latente e o tratamento com isoniazida (INH). Gostaríamos ainda de saber sua opinião sobre o acompanhamento dos adultos e crianças que vivem com um paciente com TB ativa (doença). Isso não é um teste; não existem respostas certas ou erradas. Sabemos que o seu tempo é limitado e somos gratos por sua contribuição. Não usaremos mais que 10 -15 minutos do seu tempo para responder a este questionário.* | |
| **Informações gerais sobre você** | |
| 1. | Há quanto tempo você trabalha nesta unidade de saúde?  |__|__| anos e |__|__|meses |
| 2. | Você teve algum treinamento para TB nos últimos 10 anos?  |__| Sim |__| Não **(Se não, vá para a pergunta 4**) |
| 2a. | Se sim, há quanto tempo foi o seu último treinamento (mais recente)?  |__|__| anos |
| 3. | Se sim, que tipo de treinamento foi? (Pode marcar mais de uma opção)  |__| aconselhamento de pacientes  |__| atendimento a pacientes  |__| busca de pessoas sintomáticas respiratórias  |__| tratamento da TB  |__| atendimento dos contatos  |__| causa da doença  |__| prevenção da doença  |__|Como coletar material para exame de escarro  |__| Outros (Especifique)_______________________________________ |
| 4. | Quais são as atribuições e orientações que os ACS realizam aos pacientes com TB e seus contatos aqui nesta unidade?  |__| Como tomar a medicação (hora do dia, com ou sem alimentação, etc.)  |__| Por quanto tempo deverá tomar a medicação  |__| Quando deverá retornar ao setor  |__| Como proteger outros membros da sua família da infecção  |__| O que fazer se algum efeito colateral/intolerância ocorrer  |__| Trazer todas as pessoas que vivem com ele para serem avaliadas  |__| Trazer apenas as crianças que vivem com ele para serem avaliadas  |__| Parar de fumar (se for importante)  |__| Parar de beber (se for importante)  |__| Informações sobre alimentação  |__| Não é atribuição da minha profissão  ______________________________________________________________________ |

| **Agora vamos fazer perguntas sobre o que você sabe sobre a tuberculose** | |
| --- | --- |
| **Agora vamos fazer perguntas sobre o que você sabe sobre a tuberculose** | |
| 5. | Existe TB doença e TB infecção (ou TB latente). Você sabe quais são as diferenças?  |__|Não sabe a diferença  |__| A pessoa com TB tosse  |__| A pessoa com TB tem sintomas e com infecção não tem.  |__| A pessoa com TB tem febre  |__| A pessoa com TB perde peso  |__| A pessoa com TB latente é assintomática  |__| Outros  ______________________________________________________________________________________________________________________________________________ |
| ***Nesse momento, o entrevistador deve explicar a diferença entre TB ativa e TB infecção (ILTB)*** | |
| 6. | Como se pode afirmar que a pessoa está infectada com o bacilo da tuberculose?  |__| Pessoa com tuberculose pulmonar assintomática  |__| Pessoa com tuberculose extrapulmonar assintomática  |__| Pessoa com prova tuberculínica (PPD) ou IGRA positivo  |__| Pessoa assintomática  |__| Pessoa com Rx de tórax normal  |__| Pessoa com prova tuberculínica (PPD) ou IGRA positivo e Rx de tórax normal  |__| Pessoa assintomática com prova tuberculínica (PPD) ou IGRA positivo e Rx de tórax normal  |__|Pessoa com exame de BAAR (ou de escarro ou baciloscopia) positivo  |__| Eu não sei  |__| Outros (Especifique)  ___________________________________________________________________________________________________________________________________________________________________________________________________________ |
| 7. | Como se previne que um contato (pessoa que mora com paciente com diagnóstico recente de TB ativa) venha a se infectar com TB?  |__| Dormir em uma cama diferente da do paciente com TB  |__| Não ficar muito perto do caso índice  |__| Caso índice deve usar máscara ou lenço quando tossir  |__| Descansar e não fazer esforço  |__| Não compartilhar utensílios de alimentação com o paciente com TB  |__| Não compartilhar escova de dente com o paciente com TB  |__| Parar de fumar  |__| Parar de consumir bebida alcoólica  |__| Comer melhor  |__| Tomar a vacina BCG  |__| Tomar isoniazida ou outro tratamento para infecção latente (quimioprofilaxia)  |__| Manter a casa ventilada  |__| Tomar vitaminas  |__| Não sei  |__|Hábitos de higiene  |__|Acompanhamento médico  |__| Outros  ____________________________________________________________________________________________________________________________________________________________________________________________________________________________________________________________________________________________ |
| 8. | Como se previne que uma pessoa uma vez infectada venha a adoecer?  |__| Dormir em uma cama diferente da do paciente com TB  |__| Não ficar muito perto do caso índice  |__| Caso índice deve usar máscara ou lenço quando tossir  |__| Descansar e não fazer esforço  |__| Não compartilhar utensílios de alimentação com o paciente com TB  |__| Não compartilhar escova de dente com o paciente com TB  |__| Parar de fumar  |__| Parar de consumir bebida alcoólica  |__| Comer melhor  |__| Tomar a vacina BCG  |__| Tomar isoniazida ou outro tratamento para infecção latente (quimioprofilaxia)  |__| Manter a casa ventilada  |__| Tomar vitaminas diariamente  |__|Com medicamentos (não sabe dizer qual)  |__|Acompanhamento médico semanal  |__| Não sei  |__| Outros  ____________________________________________________________________________________________________________________________________________________________________________________________________________________________________________________________________________________________ |
| 9. | Esta pergunta só deve ser feita se houver PPD ou QFT disponível na rede |
|  | De acordo com as recomendações do PNCT, que contatos intradomiciliares devem receber o tratamento para prevenção da TB?  |__| Todos  |__| Todos, desde que TB ativa seja afastada  |__| Todos sem TB ativa e com PPD ou QFT**+**  |__| Todos os menores de 15 anos  |__| Todos menores de 15 anos, desde que TB ativa seja afastada  |__| Menores de 15 anos, sem TB ativa e com PPD ou QFT**+**  |__| Todos os menores de 5 anos  |__|Todos menores de 5 anos, desde que TB ativa seja afastada  |__| Menores de 5 anos, sem TB ativa e com PPD ou QFT**+**  |__| A prevenção não é recomendada no Brasil  |__| Eu não sei  ___________________________________________________________________________________________________________________________________________________________________________________________________________________________________________________________________________________________________________________________________________________________________ |
| 9a. | Esta pergunta só deve ser feita se não houver PPD ou QFT disponível na rede |
|  | De acordo com as recomendações vigentes do PNCT, na ausência de PPD disponível, que contatos intradomiciliares devem receber o tratamento para prevenção da TB?  |__| Todos  |__| Todos, desde que TB ativa seja afastada  |__| Todos os menores de 15 anos  |__| Todos menores de 15 anos, desde que TB ativa seja afastada  |__|Todos menores de 5 anos, desde que TB ativa seja afastada  |__| Menores de 5 anos, sem TB ativa  |__| A prevenção não é recomendada no Brasil  |__| Eu não sei  |__|Outros__________________________________________________________________________________________________________________________________________________________________________________________________________________________________________________________________________________________________________________________________________________________ |

| **Agora queremos conhecer sua opinião sobre prevenção da TB doença e TB latente** | |
| --- | --- |
| *Nesta parte do questionário estamos interessados, apenas, na sua opinião. Não há respostas certas ou erradas e nós não iremos divulgar sua opinião* | |
| 10. | Você acha que é importante que uma criança que mora com um paciente com TB ativa seja investigada para TB ativa?  |__| Sim |__| Não |__| Não sei |
| 11. | Você acha que é importante que uma criança que mora com um paciente com TB ativa seja investigada para TB latente?  |__| Sim |__| Não |__| Não sei |
| 12. | Você acha que é importante que um adulto que mora com um paciente com TB ativa seja investigado para TB ativa?  |__| Sim |__| Não |__| Não sei |
| 13. | Você acha que é importante que um adulto que mora com um paciente com TB ativa seja investigado para TB latente?  |__| Sim |__| Não |__| Não sei |
| 14. | Você acha que a unidade de saúde na qual trabalha deve ser responsável pela investigação de contatos que moram com um paciente com TB ativa, ou devem fazer em outro local?  |__| Investigar crianças, mas adultos não  |__| Investigar adultos, mas crianças não  |__| Investigar adultos e crianças  |__| Não Investigar nem adultos nem crianças, e encaminhá-los para outro lugar  |__| Não é necessário investigá-los  |__| Outros  ____________________________________________________________________________________________________________________________________________________________________________________________________________________________________________________________________________________________ |
| 15. | Quais são as dificuldades **desta clínica** para avaliar um contato que vive com um paciente com TB doença?  |__| Não temos nenhuma dificuldade para investigar contatos aqui  |__| Somos muito ocupados na nossa unidade de saúde  |__| Eu não fui treinado adequadamente para avaliar contatos  |__| Pode-se causar resistência aos medicamentos tratando contatos apenas com INH  |__| Os métodos de investigação para TB nesta unidade de saúde não são bons  |__|Não temos Rx  |__| Isso não é uma prioridade  |__| Não acredito que o tratamento reduza o risco de adoecer  |__| Tenho medo dos efeitos colaterais  |__| A carga extra de trabalho me deixa estressado  |__| Eu não me sinto seguro quando estou avaliando contatos para TB ativa  |__|Todas as crianças são vacinadas pela BCG e não precisam de outras prevenções  |__| Eu deveria ser treinado para fazer o PPD  |__| Os contatos não aparecem na unidade de saúde  |__|A falta do PPD  |__|O material coletado para exame de “escarro” demora a ser enviado ao laboratório.  |__| Outros  ____________________________________________________________________________________________________________________________________________________________________________________________________________________________________________________________________________________________ |
| 16. | Algumas vezes os pais/responsáveis pelas crianças podem não trazê-las para a investigação. Quando isso acontece, o que vc acha que são as principais razões?  |__|Eles sempre trazem  |__|Eles não entendem o quanto é importante a investigação das suas crianças  |__| Eles não têm condições financeiras para levar as crianças para serem investigadas  |__| Eles só vêm à unidade de saúde quando as crianças estão doentes  |__| Eles preferem levar as crianças para serem investigadas em outra  unidade de saúde  |__| Eles têm preguiça de levar as crianças para serem investigadas  |__| Eles não são orientados a trazer as crianças para serem investigadas  |__| Eles não comparecem por causa do preconceito com a TB  |__|Crenças e/ou outros tratamentos religiosos  |__|O caso índice e/ou responsáveis não têm condições de trazer as crianças pois também estão doentes.  |__| Eu não sei  |__| Outros  __________________________________________________________________________________________________________________________________________________________________________________________________________________________________________________________________________________________________________________________________________________________________ |

| 17. | E os adultos? Algumas vezes os contatos adultos não vêm à unidade para serem investigados.  Quais vc acha que são as principais razões?  |__| Eles sempre vêm  |__| Eles não entendem o quanto é importante esta investigação  |__| Eles não têm condições financeiras para ir à unidade de saúde  |__| Adultos só vêm a clinica quando estão doentes  |__| Eles vão a outras unidades ou hospital para serem investigados  |__| Adultos tem preguiça de ir à unidade para serem investigados  |__| Os adultos não são orientados a vir à unidade de saúde para serem investigados  |__| Adultos tem medo de vir por causa do preconceito contra a TB  |__| não têm tempo de vir à unidade, precisam trabalhar  |__| Eu não sei  |__| Outros  ____________________________________________________________________________________________________________________________________________________________________________________________________________________________________________________________________________________________ |
| --- | --- |
|  |  |
| **Agora queremos saber o que você faz na prática com contatos intradomiciliares de pacientes com TB (independentemente das recomendações)** | |
| 18. | O que você faz para um adulto, contato de um paciente que vive na mesma casa que teve um diagnóstico recente de TB?  |__| Encaminho para o médico ou enfermeiro  |__| Pergunto se tem algum sintoma  |__| Encaminho para fazer PPD  |__| Colho escarro  |__| Não faço nada  |__| Não sei  |__| Outros  ____________________________________________________________________________________________________________________________________________________________________________________________________________________________________________________________________________________________ |
| 19. | E com as crianças? O que você faz para uma criança, contato de um paciente que vive na mesma casa que teve um diagnóstico recente de TB?  |__| Encaminho para o médico ou enfermeiro  |__| Pergunto se tem algum sintoma  |__| Encaminho para fazer PPD  |__| Colho escarro  |__| Não faço nada  |__| Não sei  |__| Outros  ____________________________________________________________________________________________________________________________________________________________________________________________________________________________________________________________________________________________ |
| 20. | O que você faz se uma criança em uso de isoniazida para o tratamento da TB latente tiver algum efeito colateral ao remédio, do tipo enjoo? (marque apenas uma opção)  |__| Encaminho para o médico ou enfermeiro  |__|Encaminho para uma unidade especializada  |__| Mando insistir com o tratamento  |__| Mando parar o tratamento  |__| Eu não sei  |__| Outros  ____________________________________________________________________________________________________________________________________________________________________________________________________________________________________________________________________________________________ |
| 21. | E com o adulto? O que você faz para um adulto, em tratamento para a TB latente com isoniazida que tem algum efeito colateral do tipo enjoo?  |__| Encaminho para o médico ou enfermeiro  |__| Mando insistir com o tratamento  |__| Mando parar o tratamento  |__| Eu não sei  |__| Outros  ____________________________________________________________________________________________________________________________________________________________________________________________________________________________________________________________________________________________ |
| 22. | O que você faz se uma criança em uso de isoniazida para o tratamento da TB latente ficar amarela? (marque apenas uma opção)  |__| Encaminho para o médico ou enfermeiro  |__| Mando insistir com o tratamento  |__| Mando parar o tratamento  |__| Eu não sei  |__| Outros  ____________________________________________________________________________________________________________________________________________________________________________________________________________________________________________________________________________________________ |
| 23. | E um adulto? O que você faz se um adulto em uso de isoniazida para o tratamento da TB latente ficar amarelo? (marque apenas uma opção)  |__| Encaminho para o médico ou enfermeiro  |__| Mando insistir com o tratamento  |__| Mando parar o tratamento  |__| Eu não sei  |__| Outros  ____________________________________________________________________________________________________________________________________________________________________________________________________________________________________________________________________________________________ |
| ***Chegamos ao final do nosso questionário. Obrigada por ter respondido.*** | |
| **Horário de encerramento da entrevista |__|__|:|__|__|** | |
